# Whole Genome Sequencing and Extracellular Metabolite Profiling of *Lactiplantibacillus plantarum* FRT4: Insights into Probiotic Functionality

**DOI:** 10.3390/biology14091167

**Published:** 2025-09-01

**Authors:** Yuyin Huang, Kun Meng, Guohua Liu, Zhimin Chen, Yunsheng Han, Peilong Yang, Rui Zhang, Hongying Cai

**Affiliations:** 1 Key Laboratory of Feed Biotechnology of Ministry of Agriculture and Rural Affairs, Institute of Feed Research, Chinese Academy of Agricultural Sciences, Beijing 100081, China; yuyinhuang99@163.com (Y.H.); mengkun@caas.cn (K.M.); liuguohua@caas.cn (G.L.); chenzhimin@caas.cn (Z.C.); hanyunsheng@caas.cn (Y.H.); yangpeilong@caas.cn (P.Y.); 2 Key Laboratory of Yunnan for Biomass Energy and Biotechnology of Environment, Yunnan Normal University, Kunming 650500, China

**Keywords:** *Lactiplantibacillus plantarum* FRT4, whole genome sequencing, untargeted metabolomics, probiotic, extracellular metabolites, amino acid metabolism, functional foods, strain-specificity

## Abstract

*Lactiplantibacillus plantarum* is a widely studied probiotic species with significant strain-specific functional diversity, yet the molecular mechanisms underlying these variations remain largely unexplored. In this study, whole genome sequencing (WGS) and untargeted metabolomics were employed to comprehensively characterize the genetic architecture and extracellular metabolic profile of *Lp. plantarum* FRT4 (CGMCC 17955), a probiotic strain previously studied for its metabolic effects in animal models. WGS revealed a circular chromosome and five plasmids, encoding 3301 protein-coding genes enriched in amino acid biosynthesis, carbohydrate metabolism, and environmental response pathways. Carbohydrate-active enzymes (CAZy) annotation revealed 135 carbohydrate-active enzyme genes, dominated by glycoside hydrolases and glycosyl transferases. Untargeted metabolomic analysis comparing the fermentation supernatant of FRT4 with non-inoculated MRS medium revealed significant alterations in metabolite composition, including elevated levels of acetylcholine, nicotinamide adenine dinucleotide (NAD), and trans-3-coumarate, and reduced levels of uridine, inosine, and fructose-1-phosphate, indicating active modulation of neurotransmission, redox balance, and purine metabolism. KEGG (Kyoto Encyclopedia of Genes and Genomes) enrichment showed significant regulation of pathways related to amino acid metabolism, carbon metabolism, and cofactor biosynthesis. These findings highlight the metabolic versatility and functional potential of FRT4, offering mechanistic insights into its probiotic effects and providing a basis for its potential application in fermentation-based formulations.

## Simple Summary

The microbes in the gut can influence many aspects of health, including weight, digestion, and immune function. One type of helpful microbe, *Lactiplantibacillus plantarum* FRT4, was studied to understand how it might support better health. We examined its full genetic information and measured the compounds it produces during fermentation. We discovered that this strain contains many genes related to nutrient processing and produces various beneficial compounds, including some linked to brain function and immune support. Additionally, it releases a wide variety of compounds linked to brain signaling, antioxidant activity, and gut balance, such as acetylcholine and nicotinamide adenine dinucleotide (NAD). These changes suggest the strain actively shapes its fermentation environment through metabolic activity. The findings provide molecular insights into the biosynthetic capacity of FRT4 and may inform future studies on its use in fermentation processes. This research supports its potential use in functional food development and offers a basis for further investigation into strain-specific metabolic properties.

## 1. Introduction

In recent years, the gut microbiota has attracted increasing attention for its vital role in maintaining host health, particularly in relation to metabolic disorders, immune regulation, and neural signaling [1,2,3,4,5,6]. Probiotics, as an effective strategy for modulating the composition and function of the gut microbiota, have been widely applied in both the food industry and clinical interventions [7,8,9]. Among them, lactic acid bacteria (LAB) probiotics are especially notable for their high safety profile and robust metabolic activity, contributing significantly to intestinal homeostasis, inflammatory regulation, and the improvement of metabolic imbalances [9,10,11,12]. *Lactiplantibacillus plantarum* (formerly *Lactobacillus plantarum*) is a LAB species commonly found in fermented foods and the human gut, and plays a pivotal role in probiotic research and development [13]. Studies have demonstrated that *Lp. plantarum* can modulate gut microbiota, alleviate inflammation, and mitigate both neurological and metabolic disorders, underscoring its strong potential in the development of functional foods and therapeutic strategies for metabolic diseases [14,15,16,17,18]. In addition, various strains of *Lp. plantarum* have demonstrated key probiotic features, including acid and bile tolerance, auto-aggregation, surface hydrophobicity, hydrogen peroxide production, epithelial adhesion, and antimicrobial activity, highlighting its diverse probiotic attributes [19,20,21,22].

Although *Lp. plantarum* exhibits broad probiotic potential at the species level, its physiological functions and metabolic characteristics are highly strain-specific [23]. A study by Liu et al. [24] demonstrated that different *Lp. plantarum* strains exhibit significant strain-specific anti-inflammatory effects in both Caco-2 cells and DSS-induced animal models, which are closely associated with their genetic characteristics. Similarly, research by Ramos et al. [25] showed that *Lp. plantarum* strains isolated from Brazilian foods display diverse probiotic properties that are strain-specific rather than species-specific. Strains from different sources and genetic backgrounds vary substantially in terms of metabolite production, environmental adaptability, and host interaction mechanisms [23]. Therefore, relying solely on phenotypic observations or animal experiments is insufficient to comprehensively evaluate the functional potential and underlying mechanisms of a specific strain. In recent years, the rapid advancement of high-throughput sequencing and metabolomics technologies has enabled an integrated approach combining whole-genome sequencing (WGS) and metabolomics, offering a powerful tool for probiotic research [26]. By systematically analyzing both the genetic potential and actual metabolic outputs, this strategy allows for the elucidation of strain-specific metabolic networks and associated regulatory pathways. Such insights provide molecular-level evidence for probiotic mechanisms and offer a theoretical basis for the screening and application of functional strains.

The *Lp. plantarum* FRT4 is a strain with previously reported metabolic effects in animal models, including alleviation of high-fat diet-induced weight gain, fat accumulation, hyperlipidemia, and hepatic steatosis [27,28,29,30]. Additionally, it exhibits strong protective effects on the intestinal barrier and modulates gut microbiota composition effectively [28,30]. However, despite these promising findings at the phenotypic and metabolic levels, the molecular basis underlying the probiotic potential of the FRT4 strain remains largely unclear. To date, there has been a lack of systematic investigation into its genomic architecture and metabolic regulatory networks, and their relationships with its functional mechanisms. This knowledge gap limits the further development and precise application of FRT4 in probiotic-based interventions and functional food formulations.

Therefore, in this study, we employed an integrated approach combining WGS and untargeted metabolomics to systematically evaluate the metabolic regulatory potential of FRT4 at both the gene-encoded functional level and the extracellular metabolite profile. This study aims to uncover the underlying probiotic mechanisms of the FRT4 strain and provide molecular evidence to support its functional development. These findings are expected to enhance our molecular understanding of FRT4, support its future application in functional food research, and provide a foundation for further functional validation.

## 2. Materials and Methods

### 2.1. Preparation of Lp. plantarum FRT4 Strain and Its Supernatant

As previously described [29], *Lp. plantarum* FRT4 was isolated from a type of yogurt in Xinjiang Province, China, and is preserved at the Chinese General Microorganism Collection Center under accession number 17955. The strain was cultured using the de Man–Rogosa–Sharpe (MRS) medium. The MRS formulation used in this study was as follows (per liter): 20 g glucose, 5 g peptone, 4 g yeast extract, 5 g beef extract, 10 g tryptone, 2 g diammonium hydrogen citrate, 5 g sodium acetate, 2 g dipotassium phosphate (K_2_HPO_4_), 0.5 g magnesium sulfate (MgSO_4_), 0.05 g manganese sulfate (MnSO_4_), and 1.0 mL Tween 80; the pH was adjusted to 6.5 prior to sterilization. The MRS medium was procured from Beijing Solarbio Science & Technology Co., Ltd. (Beijing, China). FRT4 was stored at −80 °C in a 1:1 (*v*/*v*) mixture of cell culture and 50% glycerol. Before use, the strain was first activated in MRS broth at 37 °C for 24 h, followed by subculturing using a 2% inoculum for 48 h. After centrifugation, both the bacterial pellet and the cell-free supernatant were collected for WGS and metabolomic analysis, respectively. As a control (CT group) for the metabolomics experiment, plain MRS broth without bacterial inoculation was subjected to the same two-step incubation process at 37 °C, followed by centrifugation to collect the supernatant.

### 2.2. Genomic DNA Extraction

Genomic DNA was extracted from the bacterial sample FRT4 using the QIAGEN Genomic-tip 20/G kit (QIAGEN, Cat 10223, Hilden, Germany), following the manufacturer’s protocol. Briefly, bacterial cells were harvested and ground in liquid nitrogen using a pre-cooled sterile mortar and pestle. Each sample (~250 mg) was lysed in Buffer G2 and incubated at 50 °C, followed by centrifugation at 4 °C to collect the supernatant. The lysate was loaded onto the equilibrated Genomic-tip column and washed with Buffer QC. DNA was eluted with Buffer QF, precipitated with isopropanol, and washed twice with 75% ethanol. The pellet was air-dried and dissolved in TE buffer. The concentration and purity of the DNA were assessed using Nanodrop 2000 (Thermo Fisher Scientific, Waltham, MA, USA) and Qubit 3.0 (Invitrogen, Waltham, MA, USA). The DNA concentration was 86.4 ng/μL, and the total yield was 3.8016 μg in 44 μL. The optical absorbance ratios were OD260/280 = 1.86 and OD260/230 = 2.38, indicating high purity suitable for long-read sequencing. DNA integrity was confirmed by agarose gel electrophoresis.

### 2.3. Whole Genome Sequencing and Library Preparation

Whole-genome sequencing and subsequent bioinformatic analysis were performed by Biomarker Technologies Co., Ltd. (Beijing, China). High-quality genomic DNA was used to construct sequencing libraries with the Oxford Nanopore SQK-LSK109 Ligation Sequencing Kit and Native Barcoding Kit SQK-NBD114.24. DNA (2 μg) was fragmented using Covaris g-TUBE (Cat 520079), followed by end-repair and dA-tailing using the NEBNext FFPE DNA Repair Mix and Ultra II End-Prep Enzyme Mix. Barcode ligation was performed with Blunt/TA Ligase Master Mix, and the final sequencing adapter was ligated using Quick T4 DNA Ligase. All purification steps were conducted using AMPure XP beads (Beckman Coulter, Brea, CA, USA). Final libraries were quantified using Qubit and adjusted to 30 ng/μL.

### 2.4. Sequencing and Assembly

Sequencing of the *Lp. plantarum* FRT4 genome was carried out on the Oxford Nanopore PromethION 48 platform using a FLO-PRO002 flow cell, with sequencing runs performed for 72 h. Raw data were base-called using Guppy v3.2.6 to generate FASTQ-formatted reads. Reads shorter than 2000 bp or of low quality were filtered out, and the high-quality reads were then assembled de novo using Canu v1.5 [31]. The draft genome was subsequently polished with Racon v3.4.3 and circularized using Circlator v1.5.5. To further improve assembly accuracy, Pilon v1.22 was used for error correction. The final assembly yielded six contigs, including one circular chromosome and five plasmids, with a total genome length of 3,449,239 bp and a coverage depth of approximately 337.63× (calculated as 1,164,567,231 bp total bases/3,449,239 bp genome size).

### 2.5. Genome Annotation and Bioinformatic Analysis

Coding sequences (CDSs) were predicted using Prodigal v2.6.3 [32]. Repetitive sequences were identified using RepeatMasker v4.0.5 [33] with a reference repeat sequence database. Non-coding RNAs, including tRNAs and rRNAs, were predicted using tRNAscan-SE v2.0 [34] and Infernal v1.1.3 [35], respectively. CRISPR arrays were identified using CRT v1.2 [36]. Genomic islands were predicted with IslandPath-DIMOB v0.2 [37], while prophage regions were detected using PhiSpy v2.3 [38]. Secondary metabolite biosynthetic gene clusters were identified through antiSMASH v5.0.0 [39]. Circular genome maps were generated using Circos v0.66 [40].

Functional annotation of protein-coding genes was carried out using sequence alignment against multiple public databases, including KEGG, Gene Ontology (GO), and eggNOG. Special functional annotations were performed using the Carbohydrate-Active Enzymes (CAZy) database for carbohydrate-active enzymes, the Comprehensive Antibiotic Resistance Database (CARD) database for antibiotic resistance genes (via RGI), and the Virulence Factor Database (VFDB) for virulence factor genes. Genome structure visualization was conducted using Circos v0.66 to produce circular genome maps integrating gene annotations, GC content, GC skew, and other features. The analyses were performed using BMKCloud (www.biocloud.net, accessed on 1 July 2025).

### 2.6. Untargeted Metabolomics Analysis

Bacterial culture supernatants were subjected to untargeted metabolomic analysis by Biotree Biotech Co., Ltd. (Shanghai, China). For each sample, 100 μL was mixed with 400 μL of extraction solution (methanol: acetonitrile = 1:1, *v*/*v*) containing deuterated internal standards. The mixture was vortexed for 30 s, sonicated for 10 min in an ice-water bath, and incubated at −40 °C for 1 h to precipitate proteins. Samples were then centrifuged at 12,000 rpm (13,800× *g*, radius = 8.6 cm) for 15 min at 4 °C. The supernatant was transferred to LC-MS vials for analysis. A quality control (QC) sample was prepared by pooling equal volumes of each sample extract from all experimental groups. This pooled QC sample was used to represent the overall metabolic composition of the study and to monitor the stability and reproducibility of the LC-MS system throughout the analysis. Metabolite separation was performed on a Vanquish UHPLC system (Thermo Fisher Scientific) using a Waters Acquity UPLC BEH Amide column (2.1 mm × 50 mm, 1.7 μm). The mobile phase consisted of solvent A (25 mmol/L ammonium acetate and 25 mmol/L ammonium hydroxide in water, pH = 9.75) and solvent B (acetonitrile). The autosampler was maintained at 4 °C, and the injection volume was 2 μL. Mass spectrometry was conducted on an Orbitrap Exploris 120 (Thermo Fisher Scientific) equipped with an electrospray ionization (ESI) source operating in both positive and negative ion modes. MS1 resolution was set to 60,000 and MS/MS resolution to 15,000. The spray voltage was 3.8 kV (positive) or −3.4 kV (negative); capillary temperature was 320 °C; sheath gas and auxiliary gas were set to 50 and 15 arbitrary units, respectively. Collision energies were set at 20/30/40 (stepped). Raw data were converted to mzXML format using ProteoWizard (v3.0) and processed using an in-house R-based program (XCMS kernel, v3.6.2) for peak detection, alignment, and integration. The annotation method of metabolites is consistent with the KGMN framework implemented in MetDNA2 [41]. Metabolite annotation was performed using the BiotreeDB (V2.1) MS/MS spectral database with a similarity score cutoff of 0.3.

Statistical analysis was conducted using SIMCA (v16.0.2) and R (v4.2.3). For multivariate analysis, orthogonal partial least squares discriminant analysis (OPLS-DA) was performed to obtain the variable importance in projection (VIP) for each metabolite. For univariate statistical testing, pairwise comparisons between groups (*N* = 5 per group) were conducted using two-tailed Student’s *t*-tests. Prior to hypothesis testing, variance homogeneity was assessed for each metabolite. When variances were unequal, Welch’s *t*-test was applied to ensure robustness. Normality tests were not performed for each metabolite due to the limited sample size. Peak quantification was based on peak area (area under the curve, AUC), and missing values were imputed using half of the minimum value across the dataset. All samples were normalized using internal standard (IS)-based normalization to correct for technical variation and batch effects. Significantly differential metabolites were defined as those with *p* < 0.05 and VIP > 1. Volcano plots were generated using R (ggplot2 v3.3.5) based on all detected metabolite features, not only the significantly changed ones. Each point in the plot represents a single metabolite feature, with the x-axis showing the log_2_-transformed fold change and the y-axis showing the –log_10_-transformed *p*-value. The point size reflects the VIP score, and point color indicates the regulation direction (upregulated, downregulated, or non-significant). This visualization strategy provides a comprehensive view of the overall metabolite distribution and differential patterns. Identified metabolites were annotated using the KEGG (Kyoto Encyclopedia of Genes and Genomes) database by mapping their KEGG compound IDs to metabolic pathways, and pathway enrichment analysis was performed to explore biological significance.

## 3. Results

### 3.1. Genomic Features of Lp. plantarum FRT4

**Figure 1:**
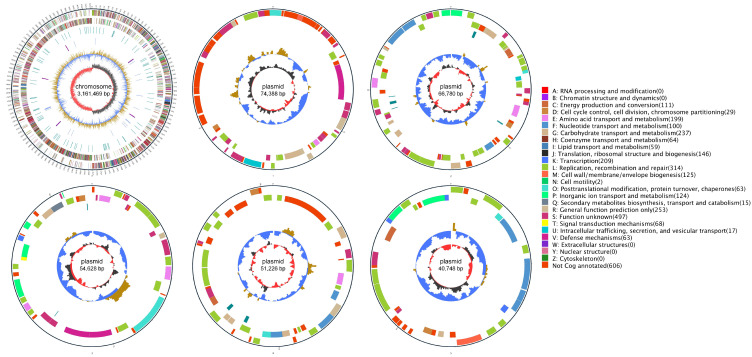
Genome map of *Lp. plantarum* FRT4, generated using Circos v0.66. The genome consists of one circular chromosome (3,161,469 bp) and five plasmids (74,388 bp, 66,780 bp, 54,628 bp, 51,226 bp, and 40,748 bp), with a total genome size of 3,449,239 bp and a G+C content of 44.27%. The outermost circle denotes genome size, with scale marks at 5 kb intervals. The second and third circles display genes encoded on the forward and reverse strands, respectively, with different colors representing distinct COG functional categories. The fourth circle indicates repetitive sequences. The fifth circle marks tRNA (blue) and rRNA (purple) regions. The sixth circle shows GC content, where light yellow denotes regions with GC content above the genomic average (higher peaks reflect greater deviations), while blue indicates below-average GC content. The innermost circle represents GC-skew, with dark gray indicating regions where G > C and red where C > G.

WGS of *Lp. plantarum* FRT4 revealed a genome size of 3,449,239 base pairs (bps), with a guanine–cytosine (G+C) content of 44.27%. The circular genome map is presented in Figure 1. The genome consists of one circular chromosome and five plasmids, with sizes of 3,161,469 bp, 74,388 bp, 66,780 bp, 54,628 bp, 51,226 bp, and 40,748 bp, respectively. The plasmids contain genes involved in diverse functional categories, such as carbohydrate metabolism, replication and repair, and membrane transport, suggesting their potential roles in environmental adaptation and horizontal gene transfer. A total of 3301 protein-coding genes were predicted, spanning a cumulative length of 2,895,105 bp, accounting for 83.93% of the entire genome. The average gene length was 877 bp, with the longest gene measuring 5814 bp and the shortest 93 bp. Additionally, the genome contains 73 tRNA genes, 5 copies each of 23S rRNA and 16S rRNA, 6 copies of 5S rRNA, 15 CRISPR arrays, 14 genomic islands, 2 prophage regions, and 3 gene clusters.

### 3.2. Functional Annotation Analysis of Lp. plantarum FRT4

#### 3.2.1. Functional Annotation Based on KEGG Database

**Figure 2:**
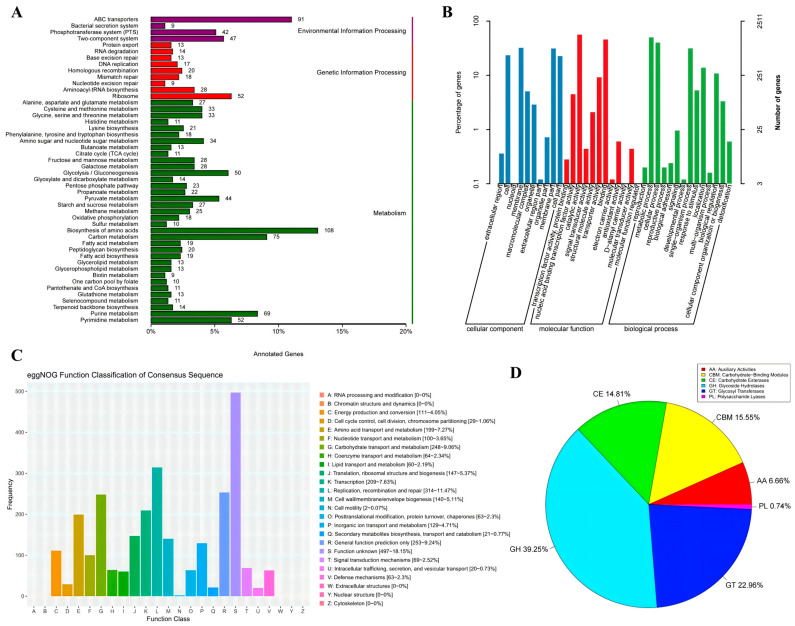
Functional annotation of WGS of *Lp. plantarum* FRT4. (**A**) KEGG functional annotation analysis. (**B**) GO database functional annotation analysis. (**C**) eggNOG database functional annotation analysis. (**D**) CAZy database functional annotation analysis.

To gain insights into the biological roles of *Lp. plantarum* FRT4, KEGG pathway annotation was performed on the predicted coding sequences (Figure 2A). A total of 1490 genes were successfully mapped to KEGG pathways, covering a broad spectrum of metabolic and cellular processes. These annotated genes were categorized into three major functional classes: metabolism, genetic information processing, and environmental information processing, with metabolism being the most enriched category.

Within the FRT4 genome annotation, 108 genes were assigned to “biosynthesis of amino acids”, which constituted the largest category among metabolic pathways, followed by “carbon metabolism” (75 genes), “purine metabolism” (52 genes), “pyruvate metabolism” (44 genes), and “glycolysis/gluconeogenesis” (50 genes). These values are in line with species-level reports for *Lp. plantarum*, which typically exhibits a robust backbone of amino acid and central carbon metabolism [13,42,43]. Other notable pathways included “amino sugar and nucleotide sugar metabolism” (34 genes), and “fructose and mannose metabolism” (28 genes), reflecting the strain’s potential for diverse carbohydrate utilization.

In the genetic information processing category, 52 genes were involved in “ribosome biogenesis”, and several others were associated with “DNA replication” (17 genes), “homologous recombination” (20 genes), and “mismatch repair” (16 genes), suggesting robust mechanisms for genome maintenance and protein synthesis.

The environmental information processing category was dominated by genes related to “ABC transporters” (91 genes), “two-component systems” (47 genes), and “phosphotransferase systems (PTS)” (42 genes), indicating that FRT4 is equipped with sophisticated signal transduction and nutrient uptake capabilities, which may contribute to its adaptability in various environments, including the gastrointestinal tract.

Collectively, these results suggest that FRT4 possesses a metabolically versatile and environmentally responsive genome, supporting its potential application as a probiotic strain with robust functional capacities.

#### 3.2.2. Functional Annotation Based on GO Database

GO annotation was conducted to further elucidate the functional roles of the predicted genes in *Lp. plantarum* FRT4 (Figure 2B). A total of 2511 genes from the FRT4 genome were successfully annotated to the GO database. The GO terms were classified into three primary categories: biological process, molecular function, and cellular component.

In the biological process category, the largest number of genes were associated with “metabolic processes”, “cellular processes”, and “biological regulation”, indicating that FRT4 harbors a wide range of genes involved in fundamental physiological and biochemical activities. Other enriched terms included “localization”, “response to stimulus”, and “signaling”, suggesting the strain’s potential to adapt to environmental changes.

Within the molecular function category, the most prominent terms included “catalytic activity” and “binding”, highlighting the strain’s enzymatic capacity and molecular interaction potential. Additionally, genes involved in “transporter activity”, “electron carrier activity”, “antioxidant activity”, and “structural molecule activity” were also identified, implying a diverse range of functional proteins.

Regarding the cellular component category, the majority of annotated genes were localized to cell parts, membranes, and macromolecular complexes, which are consistent with typical bacterial cellular architecture. Notably, a considerable number of genes were also associated with the extracellular region, which may be relevant to the strain’s probiotic properties, such as host interaction and secretion of bioactive compounds.

Overall, the GO annotation results reveal that FRT4 possesses a functionally diverse genome, with genes involved in essential biological activities, environmental responses, and cellular structures that may contribute to its probiotic potential and ecological adaptability.

#### 3.2.3. Functional Annotation Based on eggNOG Database

To gain insights into the functional distribution of coding sequences in *Lp. plantarum* FRT4, a total of 2695 genes were annotated using the eggNOG database (Figure 2C). These genes were classified into 25 functional categories based on the Clusters of Orthologous Groups (COG) system. Among them, the largest proportion of genes (497, 18.15%) were assigned to the category “function unknown,” indicating a substantial number of genes with yet uncharacterized roles. The second most abundant category was “replication, recombination and repair” (314 genes, 11.47%), followed by “carbohydrate transport and metabolism” (248 genes, 9.06%) and “general function prediction only” (253 genes, 9.24%). These results highlight the organism’s strong capacity for genome maintenance, carbohydrate utilization, and putative metabolic versatility. Other prominent categories included “amino acid transport and metabolism” (199 genes, 7.27%), “transcription” (209 genes, 7.63%), and “energy production and conversion” (111 genes, 4.05%).

Notably, a small number of genes were annotated in categories related to “secondary metabolites biosynthesis, transport and catabolism” (21 genes, 0.77%), “cell motility” (2 genes, 0.07%), and “intracellular trafficking, secretion, and vesicular transport” (20 genes, 0.73%), suggesting these functions may be limited or conditionally expressed in strain FRT4.

Overall, the functional annotation reveals that FRT4 possesses a diverse genetic repertoire, particularly enriched in genes involved in essential metabolic processes, genome stability, and nutrient transport, while also leaving room for further exploration of uncharacterized genes.

#### 3.2.4. Functional Annotation of CAZy Database

To explore the carbohydrate metabolic potential of *Lp. plantarum* FRT4, functional annotation was performed using the CAZy database. Consistent with prior *Lp. plantarum* surveys, FRT4 harbors a broad CAZyme repertoire (135 genes), supporting its versatile carbohydrate utilization [13,43,44]. The CAZyme-encoding genes were identified and classified into six major CAZy categories: glycoside hydrolases (GHs), glycosyl transferases (GTs), carbohydrate-binding modules (CBMs), carbohydrate esterases (CEs), auxiliary activities (AAs), and polysaccharide lyases (PLs) (Figure 2D; Table 1).

Among them, GHs were the most abundant family, comprising 53 genes (39.25%), highlighting the strain’s high potential for degrading complex carbohydrates through the hydrolysis of glycosidic bonds. GTs were the second most represented group, with 31 genes (22.96%), suggesting a significant capacity for glycosidic bond biosynthesis and oligosaccharide assembly. CBMs and CEs accounted for 21 (15.55%) and 20 (14.81%) genes, respectively, indicating important roles in carbohydrate substrate recognition and modification, such as deacetylation and ester bond cleavage. The AA category, associated with redox reactions and lignocellulosic biomass degradation, included 9 genes (6.66%). Only a single gene (0.74%) was assigned to the PL family, suggesting a limited role in polysaccharide cleavage via β-elimination.

These results demonstrate that FRT4 possesses a comprehensive and functionally diverse CAZyme repertoire. This enzymatic potential may contribute to the strain’s ecological versatility and its ability to metabolize a wide array of plant-derived and dietary carbohydrates, which is particularly relevant for its application in fermented foods and as a probiotic.

#### 3.2.5. Antibiotic Resistance Gene-Annotation Based on CARD

Functional annotation using CARD identified only one antibiotic resistance gene in the genome of *Lp. plantarum* FRT4. The gene was annotated as poxtA (ARO:3004470), which encodes an ABC-F subfamily ATP-binding cassette protein.

#### 3.2.6. Virulence Factor Annotation Based on the VFDB

Annotation of the *Lp. plantarum* FRT4 genome against the VFDB identified 8 genes with >60% sequence identity to known virulence factors. (Table 2).

### 3.3. Untargeted Metabolomics Reveals Significant Extracellular Metabolic Changes in FRT4 Supernatant

**Figure 3:**
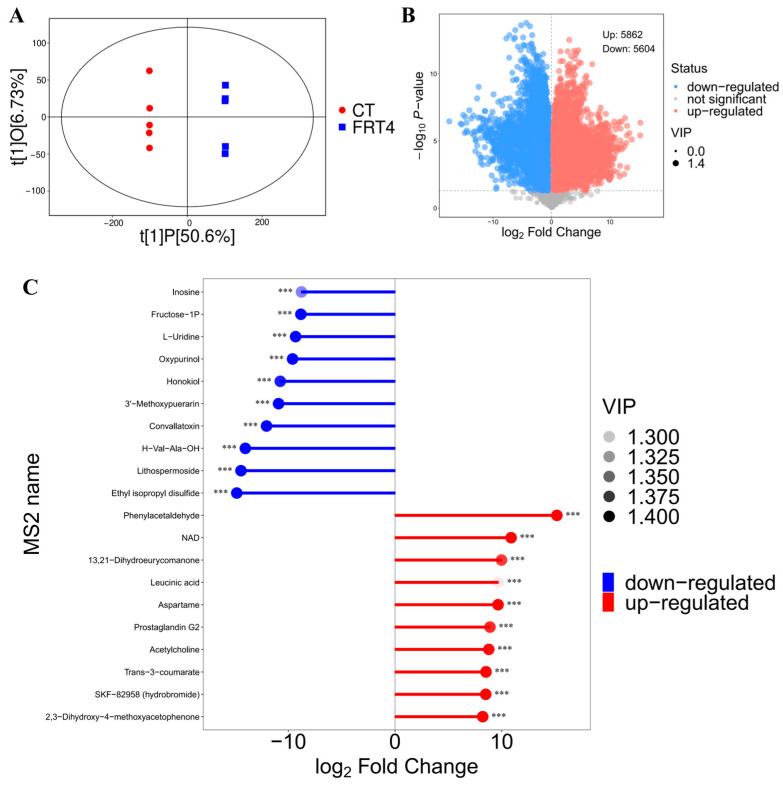
Metabolomic changes in the fermentation broth of *Lp. plantarum* FRT4. (**A**) OPLS-DA score plot. (**B**) Volcano plot. (**C**) Heatmap of differential metabolites. *N* = 5 per group. *** *p* < 0.001.

To further validate the predicted metabolic potential of *Lp. plantarum* FRT4, untargeted metabolomic profiling was performed on the fermentation supernatant. OPLS-DA analysis showed a distinct separation between the FRT4 group and the control MRS medium group (CT), indicating a significant difference in overall metabolic composition (Figure 3A).

Volcano plot analysis identified a total of 11,466 significantly altered metabolites between the FRT4 and CT groups (VIP > 1, *p* < 0.05), of which 5862 were upregulated and 5604 were downregulated in the FRT4 group (Figure 3B). These results indicate a broad metabolic reprogramming during FRT4 fermentation.

The top 20 differential metabolites were ranked by VIP score and fold change, highlighting several significantly upregulated compounds in the FRT4 supernatant, including nicotinamide adenine dinucleotide (NAD), trans-3-coumarate, acetylcholine, and prostaglandin G2. In contrast, metabolites such as inosine, fructose-1-phosphate, L-uridine, oxypurinol, and honokiol were markedly downregulated (Figure 3C). These representative metabolites indicate that FRT4 fermentation is associated with the production and transformation of compounds involved in neurotransmission, redox reactions, and nucleotide metabolism.

These findings demonstrate that *Lp. plantarum* FRT4 modifies the extracellular metabolite profile, reflecting its substrate utilization and metabolic capacity during fermentation.

### 3.4. KEGG Pathway Enrichment Analysis and Functional Implications of the Metabolome

**Figure 4:**
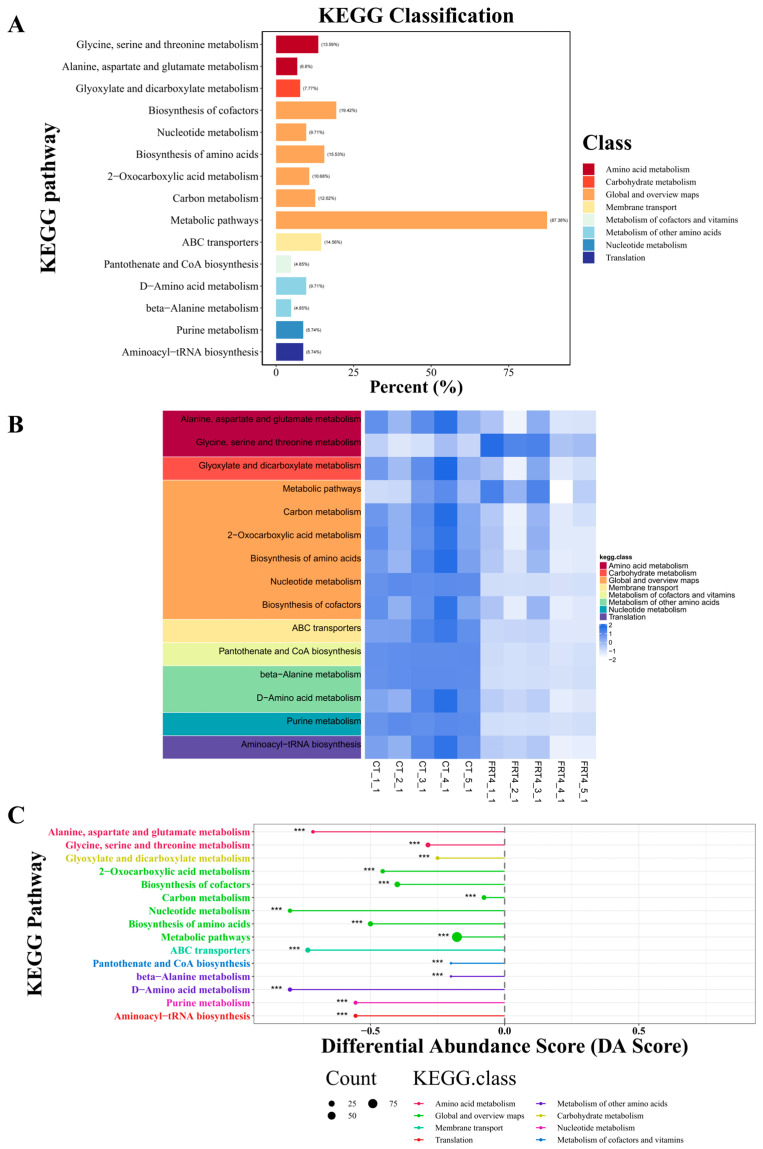
KEGG pathway analysis of the fermentation supernatant of *Lp. plantarum* FRT4. (**A**) KEGG pathway classification. (**B**) KEGG pathway heatmap. (**C**) KEGG DA score plot. *N* = 5 per group. *** *p* < 0.001.

KEGG pathway enrichment and functional classification analyses were performed to investigate the potential metabolic pathways associated with the differential metabolites during the fermentation process of FRT4. As shown in the KEGG classification plot (Figure 4A), the significantly altered metabolites were predominantly enriched in “metabolic pathways” (87.38%), “biosynthesis of cofactors” (19.42%), and “biosynthesis of amino acids” (15.53%). Additionally, the main functional categories involved fundamental metabolic activities, including amino acid metabolism (e.g., “glycine, serine and threonine metabolism” and “alanine, aspartate and glutamate metabolism”), carbohydrate metabolism (e.g., “glyoxylate and dicarboxylate metabolism”), nucleotide metabolism, and the metabolism of cofactors and vitamins.

As shown in the KEGG heatmap (Figure 4B), the abundance of metabolites involved in most key metabolic pathways was significantly lower in the FRT4 group compared to the CT group, suggesting that these metabolites may have been extensively consumed or transformed during fermentation. This trend was further confirmed by the differential abundance (DA) score analysis (Figure 4C), where most pathways exhibited negative DA scores, indicating an overall downregulation. Notably, pathways related to amino acid metabolism (such as “glycine, serine and threonine metabolism”, “alanine, aspartate and glutamate metabolism”, “biosynthesis of amino acids”, “beta-alanine metabolism”, and “D-amino acid metabolism”) were among those most significantly downregulated, reinforcing the notion that fermentation induces a systematic modulation of metabolic pathways.

**Figure 5:**
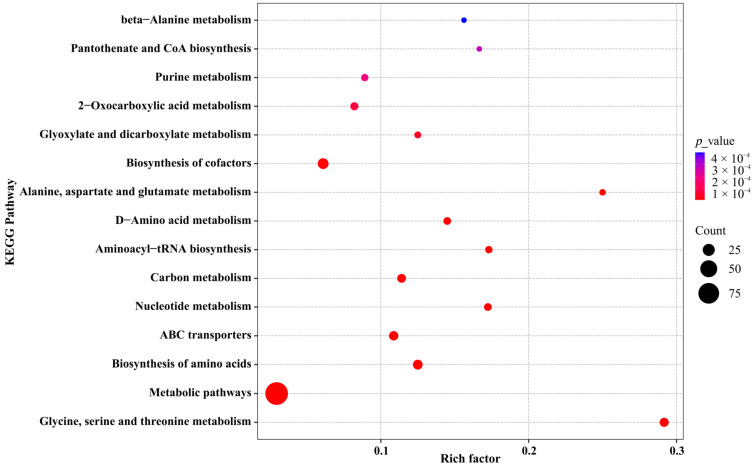
KEGG pathway enrichment bubble plot of the fermentation supernatant of *Lp. plantarum* FRT4. *N* = 5 per group.

To further assess the enrichment and statistical significance of KEGG pathways, a KEGG enrichment bubble plot was generated (Figure 5). The results revealed that the “metabolic pathways” category contained the largest number of differential metabolites and showed significant enrichment; “glycine, serine and threonine metabolism pathway” exhibited the highest enrichment factor, indicating it was most strongly affected by fermentation. Other highly enriched and statistically significant pathways included “biosynthesis of amino acids”, “D-amino acid metabolism”, “alanine, aspartate and glutamate metabolism”, “pantothenate and CoA biosynthesis”, “glyoxylate and dicarboxylate metabolism”, and “biosynthesis of cofactors”, highlighting their important roles in FRT4-mediated metabolic regulation.

**Figure 6:**
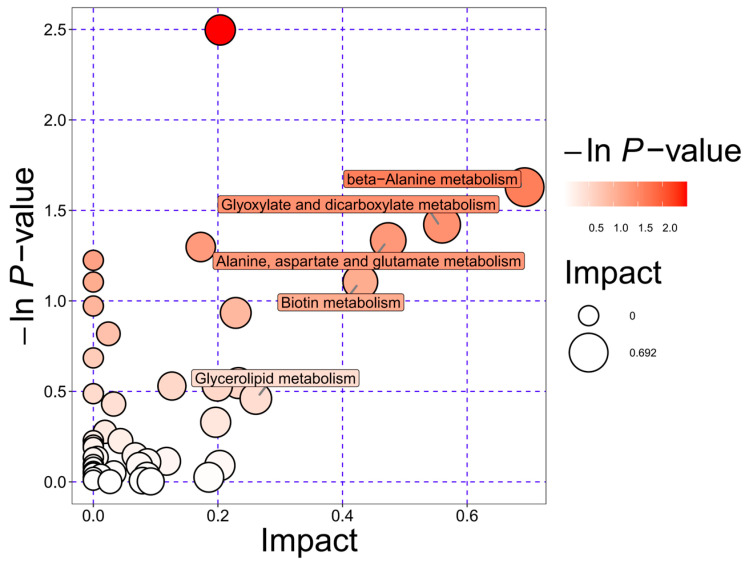
KEGG topological analysis bubble plot of the fermentation supernatant of *Lp. plantarum* FRT4. *N* = 5 per group.

Furthermore, a topological bubble plot was constructed to evaluate the functional importance of differential metabolites within each pathway (Figure 6). The x-axis represents the pathway impact value, reflecting the centrality of the altered metabolites within the metabolic network, while the y-axis indicates the statistical significance of enrichment. The results showed that “beta-alanine metabolism”, “glyoxylate and dicarboxylate metabolism”, and “alanine, aspartate and glutamate metabolism” showed significant enrichment and high impact values, suggesting that critical nodes within these pathways were significantly regulated and may play central roles in the metabolic remodeling induced by FRT4 fermentation.

Comprehensive multidimensional KEGG analyses reveal that FRT4 fermentation significantly impacts a wide range of fundamental metabolic pathways, particularly those involved in amino acid biosynthesis and degradation, carbon metabolism, and cofactor biosynthesis. These metabolic alterations may reflect the strain’s metabolic reprogramming and substrate transformation capacity during fermentation. Collectively, these findings provide metabolomic evidence of FRT4’s metabolic versatility, laying the groundwork for future studies in fermentation and functional strain screening.

## 4. Discussion

*Lp. plantarum* is a commonly found probiotic with strong environmental adaptability and metabolic versatility [45]. Previous studies have demonstrated that the FRT4 strain effectively alleviates high-fat diet-induced weight gain, fat accumulation, hyperlipidemia, and hepatic steatosis, while also improving intestinal barrier integrity, modulating gut microbiota, and regulating lipid metabolism [27,28,29,30]. However, despite phenotypic and metabolite-level evidence supporting its physiological benefits, the specific metabolic potential and molecular mechanisms underlying the physiological effects of FRT4 remain largely unclear. In this study, we systematically characterized the functional features of *Lp. plantarum* FRT4 using WGS and untargeted metabolomics. The results revealed that FRT4 encodes diverse metabolic pathways and significantly alters extracellular metabolite profiles during fermentation, indicating its strain-specific metabolic capabilities for further investigation.

The presence of a complex genome structure, including multiple plasmids, may endow FRT4 with enhanced adaptability and niche-specific functions, consistent with the typical features of *Lp. plantarum* [44]. Plasmids often carry genes involved in environmental sensing, stress responses, and horizontal gene transfer, which could explain FRT4’s resilience and functional diversity in both host and fermentation environments [42]. The enrichment of genes linked to amino acid and central carbon metabolism suggests that FRT4 is metabolically equipped to thrive in nutrient-variable environments. These findings indicate a robust substrate utilization capacity and endogenous biosynthetic potential, enabling stable and efficient metabolic activity under diverse nutritional conditions. In terms of carbohydrate metabolism, the genome of FRT4 encodes various CAZymes (e.g., GHs, GTs, and CBMs), which collectively confer the ability to efficiently degrade polysaccharides, synthesize oligosaccharides, and modify carbohydrate structures [43]. This metabolic capacity may be advantageous in plant-based or fiber-rich fermentation environments, where efficient utilization of complex carbohydrates is essential. The predicted CAZyme repertoire suggests potential for producing fermentation-derived bioactive compounds such as short-chain fatty acids and phenolic derivatives [43,46]. These features may contribute to interactions within microbial communities in fermented systems [47,48]. Furthermore, the presence of ABC transporters, two-component systems, and PTS suggests that FRT4 can dynamically sense and respond to environmental changes, aligning with its potential for metabolic adaptation in complex substrates or biotechnological settings [49,50,51,52]. Such regulatory capacity plays a key role in probiotic colonization, functional expression, and microbial interactions in dynamic environments.

To further validate the metabolic potential of FRT4 observed at the genomic level, we conducted a systematic analysis of the metabolites in its fermentation supernatant using untargeted metabolomics. The observed metabolic shift suggests that FRT4 actively transforms its extracellular environment, likely through the secretion or consumption of bioactive metabolites. Such activity reflects the metabolic activity of the strain in vitro and may be relevant to microbial interactions or fermentation dynamics. Several metabolites, including acetylcholine, NAD, and trans-3-coumarate, were enriched in the FRT4 fermentation broth. These compounds are known to play roles in neurotransmission, redox balance, and inflammation in other systems [53,54,55,56]. Their accumulation suggests that FRT4 may possess biosynthetic capacity for producing molecules of interest. While this observation is interesting, its physiological relevance remains to be explored. Meanwhile, substantial consumption of nucleotides, amino acids, and their derivatives (such as inosine and uridine) suggests broad utilization of energy and structural substrates during fermentation.

Notably, acetylcholine, a classical neurotransmitter, was markedly increased in the FRT4 fermentation broth. This molecule has been associated with gut–brain axis signaling in previous studies [57]. Previous studies have shown that probiotic strains like *Blautia wexlerae* can regulate acetylcholine levels and modulate gut microbiota, thereby improving obesity and type 2 diabetes in mice [58]. Elevated levels of NAD were also detected. As a key cofactor in energy metabolism, antioxidant defense, and DNA repair, NAD plays important roles in maintaining cellular homeostasis [59,60]. Its accumulation may reflect the active metabolic profile of *Lp. plantarum* strains. Exogenous NAD supplementation has been explored for its potential to support metabolic health and mitigate age-related dysfunctions, including obesity, diabetes, and cardiovascular conditions [61,62,63]. These findings offer new insights into the probiotic mechanisms of *Lp. plantarum* via metabolic regulation. In addition, notable reductions in several nucleotides and their derivatives, such as uridine and inosine, were observed during fermentation. This suggests active remodeling of purine and pyrimidine pathways. Beyond their role in nucleic acid synthesis, nucleotides are increasingly recognized as dynamic participants in energy metabolism and intracellular signaling, with links to disease-related metabolic processes [64,65,66]. Together, these metabolic changes highlight the robust biochemical activity of FRT4 during fermentation. While the biological relevance of individual metabolites requires further investigation, the overall profile provides a valuable basis for understanding the metabolic potential of this strain and exploring its applications in functional food development.

KEGG pathway enrichment analysis revealed that FRT4 modulated multiple core metabolic pathways during fermentation. Differentially abundant metabolites were primarily associated with amino acid metabolism, carbon metabolism, nucleotide metabolism, and cofactor biosynthesis, which are processes fundamental to cellular function. Most metabolites within these pathways were downregulated, which may reflect the active uptake and utilization of key intermediates by FRT4. In the context of carbon metabolism, FRT4 significantly influenced pathways such as “glyoxylate and dicarboxylate metabolism” and “pyruvate metabolism”. Previous studies have linked the upregulation of glyoxylate and dicarboxylate metabolism with metabolic disorders including obesity, type 2 diabetes, and atherosclerosis [67,68]. In our study, metabolites related to this pathway were reduced following fermentation, potentially indicating altered flux through these routes. While the biological implications remain to be clarified, this observation aligns with earlier findings, suggesting possible roles for *Lp. plantarum* in lipid regulation [27,28,29,30]. Pyruvate, a glycolysis product and an anaplerotic substrate for the TCA cycle, reflects the organism’s metabolic flexibility in responding to changes in fermentative substrates [69]. Additionally, several amino acid-related pathways were significantly affected, including “glycine, serine and threonine metabolism,” “alanine, aspartate and glutamate metabolism,” “D-amino acid metabolism,” and “biosynthesis of amino acids,” suggesting active amino acid uptake, transformation, and synthesis by FRT4 during fermentation. Amino acid metabolism has been implicated in various disease processes, including obesity, diabetes, and cancer [70,71,72]. The overlap between metabolomic and genomic KEGG pathway enrichment indicates consistency between metabolic output and predicted genetic capacity. This coherence suggests that FRT4 exhibits a well-aligned metabolic profile under fermentative conditions, supporting its potential relevance in applications where carbon and nitrogen balance modulation is of interest.

Building on the foregoing findings and interpretations, we also emphasize several limitations. Functional outcomes associated with the altered metabolites were not assessed experimentally in this study. Furthermore, our study did not include a cross-genome comparison positioning FRT4 among publicly available *Lp. plantarum* genomes. Such benchmarking would provide valuable species-level context and could refine the interpretation of pathway representation. Incorporating these analyses in future work would help clarify strain-specific metabolic traits and refine the interpretation of pathway functions. In addition, while FRT4 has shown promising characteristics, ongoing surveillance of its key genomic and phenotypic attributes is recommended during industrial-scale fermentation. Such monitoring would contribute to ensuring strain stability, maintaining product consistency, and safeguarding safety throughout the production process.

## 5. Conclusions

In this study, we systematically characterized the genetic features and metabolic activity of *Lactiplantibacillus plantarum* FRT4 using an integrated approach combining WGS and untargeted metabolomics. The genomic analysis revealed one circular chromosome and five plasmids, encoding over 3300 genes associated with core metabolic pathways including carbohydrate utilization, amino acid biosynthesis, and environmental adaptation. Functional annotation indicated a diverse repertoire of enzymes and transport systems, highlighting the strain’s metabolic versatility. Untargeted metabolomic analysis validated the metabolic activity of FRT4. Fermentation by FRT4 significantly altered the concentrations of various metabolites in the culture supernatant, including amino acids, nucleotides, neurotransmitters, and cofactors. Notably, elevated levels of acetylcholine, NAD, and trans-3-coumarate, as well as reduced levels of uridine, inosine, and fructose-1-phosphate, were observed. KEGG pathway enrichment analysis indicated that these differential metabolites were mainly involved in core biological processes such as “amino acid metabolism,” “carbon metabolism,” and “cofactor biosynthesis.” The findings provide molecular-level insights into the strain’s metabolic capacity during fermentation. These results may inform future investigations into the functional properties of FRT4 and support its potential application in fermentation-driven product development or microbial strain screening.

## Figures and Tables

**Table 1:** CAZyme family distribution in *Lp. plantarum* FRT4.

Type	Number	Percentage (%)
AA	9	6.66
CBM	21	15.55
CE	20	14.81
GH	53	39.25
GT	31	22.96
PL	1	0.74

**Table 2:** Prediction of virulence factors in *Lp. plantarum* FRT4.

VFDB Gene Name	VFDB Gene Function	Virulence Factor Name	Virulence Factor ID	VFDB Target ID	E-Value	Percent Identity (%)
*hasC*	UDP-glucose pyrophosphorylase	Hyaluronic acid capsule	VF0244	VFG000964	3.5572 × 10^−161^	75.34
*clpP*	ATP-dependent Clp protease proteolytic subunit	ClpP	VF0074	VFG000077	1.13222 × 10^−98^	69.79
*cpsA*	undecaprenyl diphosphate synthase	Capsule	VF0361	VFG002190	8.90991 × 10^−116^	67.51
*bsh*	bile salt hydrolase	BSH	VF0350	VFG002162	2.25858 × 10^−154^	67.11
*cpsI*	UDP-galactopyranose mutase	Capsule	VF0361	VFG002182	1.31649 × 10^−180^	65.3
*msrA/B(pilB)*	trifunctional thioredoxin/methionine sulfoxide reductase A/B protein	MsrAB	VF0456	VFG037100	5.17036 × 10^−60^	64.03
*gnd*	6-phosphogluconate dehydrogenase	Capsule	VF0560	VFG048830	0	62.87
*clpE*	ATP-dependent protease	ClpE	VF0073	VFG000080	0	60.76

## Data Availability

The original contributions presented in the study are included in the article; further inquiries can be directed to the corresponding authors. Raw data of the whole genome sequencing was deposited in the NGDC GSA database (accession number: CRA027944). Raw data of untargeted metabolomics were deposited in the NGDC OMIX database (accession number: OMIX011038).

## References

[1] Gruber T., Lechner F., Krieger J.-P., García-Cáceres C. (2025). Neuroendocrine Gut-Brain Signaling in Obesity. Trends Endocrinol. Metab..

[2] Shen Z., Cui T., Liu Y., Wu S., Han C., Li J. (2023). *Astragalus membranaceus* and *Salvia miltiorrhiza* Ameliorate Diabetic Kidney Disease via the “Gut-Kidney Axis”. Phytomedicine.

[3] Kronsten V.T., Tranah T.H., Pariante C., Shawcross D.L. (2022). Gut-Derived Systemic Inflammation as a Driver of Depression in Chronic Liver Disease. J. Hepatol..

[4] Foster J.A. (2022). Modulating Brain Function with Microbiota. Science.

[5] Ye L., Bae M., Cassilly C.D., Jabba S.V., Thorpe D.W., Martin A.M., Lu H.-Y., Wang J., Thompson J.D., Lickwar C.R. (2021). Enteroendocrine Cells Sense Bacterial Tryptophan Catabolites to Activate Enteric and Vagal Neuronal Pathways. Cell Host Microbe.

[6] Montgomery T.L., Eckstrom K., Lile K.H., Caldwell S., Heney E.R., Lahue K.G., D’Alessandro A., Wargo M.J., Krementsov D.N. (2022). *Lactobacillus reuteri* Tryptophan Metabolism Promotes Host Susceptibility to CNS Autoimmunity. Microbiome.

[7] Cho M.-Y., Eom J.-H., Choi E.-M., Yang S.-J., Lee D., Kim Y.Y., Kim H.-S., Hwang I. (2025). Recent Advances in Therapeutic Probiotics: Insights from Human Trials. Clin. Microbiol. Rev..

[8] Fentie E.G., Lim K., Jeong M., Shin J.-H. (2024). A Comprehensive Review of the Characterization, Host Interactions, and Stabilization Advancements on Probiotics: Addressing the Challenges in Functional Food Diversification. Compr. Rev. Food Sci. Food Saf..

[9] Kim M.-J., Shin S.-K., Han J.-W., Kim J.E., Lee M.J., Bae H.R., Kwon E.-Y. (2025). *Lactobacillus paragasseri* SBT2055 Attenuates Obesity via the Adipose Tissue-Muscle-Gut Axis in Obese Mice. Microbiol. Res..

[10] De Filippis F., Pasolli E., Ercolini D. (2020). The Food-Gut Axis: Lactic Acid Bacteria and Their Link to Food, the Gut Microbiome and Human Health. FEMS Microbiol. Rev..

[11] Choi Y., Park E., Kim S., Ha J., Oh H., Kim Y., Lee Y., Seo Y., Kang J., Lee S. (2021). Fermented Milk with *Lactobacillus curvatus* SMFM2016-NK Alleviates Periodontal and Gut Inflammation, and Alters Oral and Gut Microbiota. J. Dairy Sci..

[12] Gu Q., Chen Z., Liu N., Xia C., Zhou Q., Li P. (2023). *Lactiplantibacillus plantarum* ZJ316-Fermented Milk Ameliorates Dextran Sulfate Sodium-Induced Chronic Colitis by Improving the Inflammatory Response and Regulating Intestinal Microbiota. J. Dairy Sci..

[13] Echegaray N., Yilmaz B., Sharma H., Kumar M., Pateiro M., Ozogul F., Lorenzo J.M. (2023). A Novel Approach to *Lactiplantibacillus plantarum*: From Probiotic Properties to the Omics Insights. Microbiol. Res..

[14] Arellano-García L., Trepiana J., Martínez J.A., Portillo M.P., Milton-Laskibar I. (2023). Beneficial Effects of Viable and Heat-Inactivated *Lactobacillus rhamnosus* GG Administration on Oxidative Stress and Inflammation in Diet-Induced NAFLD in Rats. Antioxidants.

[15] Kumar A., Kumar V., Pramanik J., Rustagi S., Prajapati B., Jebreen A., Pande R. (2025). *Lactiplantibacillus plantarum* as a Complementary Approach for Diabetes Treatment and Management. Curr. Nutr. Rep..

[16] Duarte Luiz J., Manassi C., Magnani M., da Cruz A.G., Pimentel T.C., Verruck S. (2025). *Lactiplantibacillus plantarum* as a Promising Adjuvant for Neurological Disorders Therapy through the Brain-Gut Axis and Related Action Pathways. Crit. Rev. Food Sci. Nutr..

[17] Chen C.-M., Wu C.-C., Kim Y., Hsu W.-Y., Tsai Y.-C., Chiu S.-L. (2024). Enhancing Social Behavior in an Autism Spectrum Disorder Mouse Model: Investigating the Underlying Mechanisms of *Lactiplantibacillus plantarum* Intervention. Gut Microbes.

[18] Liang S., Sun J., Gu X., Zhao Y., Wang X., Tao H., Wang Z., Zhong Y., Wang J., Han B. (2024). *Lactobacillus plantarum* L11 and *Lactobacillus reuteri* LR: Ameliorate Obesity via AMPK Pathway. Nutrients.

[19] Stojanov S., Plavec T.V., Zupančič Š., Berlec A. (2024). Modified Vaginal *Lactobacilli* Expressing Fluorescent and Luminescent Proteins for More Effective Monitoring of Their Release from Nanofibers, Safety and Cell Adhesion. Microb. Cell Factories.

[20] Kaushik J.K., Kumar A., Duary R.K., Mohanty A.K., Grover S., Batish V.K. (2009). Functional and Probiotic Attributes of an Indigenous Isolate of *Lactobacillus plantarum*. PLoS ONE.

[21] Yadav R., Singh P.K., Puniya A.K., Shukla P. (2016). Catalytic Interactions and Molecular Docking of Bile Salt Hydrolase (BSH) from *L. Plantarum* RYPR1 and Its Prebiotic Utilization. Front. Microbiol..

[22] Rocchetti M.T., Russo P., Capozzi V., Drider D., Spano G., Fiocco D. (2021). Bioprospecting Antimicrobials from *Lactiplantibacillus plantarum*: Key Factors Underlying Its Probiotic Action. Int. J. Mol. Sci..

[23] Yu A.O., Goldman E.A., Brooks J.T., Golomb B.L., Yim I.S., Gotcheva V., Angelov A., Kim E.B., Marco M.L. (2021). Strain Diversity of Plant-Associated *Lactiplantibacillus plantarum*. Microb. Biotechnol..

[24] Liu Y., Liu Q., Zhao J., Zhang H., Zhai Q., Chen W. (2022). Strain-Specific Regulative Effects of *Lactobacillus plantarum* on Intestinal Barrier Dysfunction Are Associated with Their Capsular Polysaccharides. Int. J. Biol. Macromol..

[25] Ramos C.L., Thorsen L., Schwan R.F., Jespersen L. (2013). Strain-Specific Probiotics Properties of *Lactobacillus fermentum*, *Lactobacillus plantarum* and *Lactobacillus brevis* Isolates from Brazilian Food Products. Food Microbiol..

[26] Yang J., Shang P., Zhang B., Wang J., Du Z., Wang S., Xing J., Zhang H. (2023). Genomic and Metabonomic Methods Reveal the Probiotic Functions of Swine-Derived *Ligilactobacillus salivarius*. BMC Microbiol..

[27] Li D., Meng K., Liu G., Wen Z., Han Y., Liu W., Xu X., Song L., Cai H., Yang P. (2025). *Lactiplantibacillus plantarum* FRT4 Protects against Fatty Liver Hemorrhage Syndrome: Regulating Gut Microbiota and FoxO/TLR-4/NF-κB Signaling Pathway in Laying Hens. Microbiome.

[28] Li D., Cai H., Liu G., Han Y., Qiu K., Liu W., Meng K., Yang P. (2024). *Lactiplantibacillus plantarum* FRT4 Attenuates High-Energy Low-Protein Diet-Induced Fatty Liver Hemorrhage Syndrome in Laying Hens through Regulating Gut-Liver Axis. J. Anim. Sci. Biotechnol..

[29] Cai H., Wen Z., Xu X., Wang J., Li X., Meng K., Yang P. (2022). Serum Metabolomics Analysis for Biomarkers of *Lactobacillus plantarum* FRT4 in High-Fat Diet-Induced Obese Mice. Foods.

[30] Cai H., Wen Z., Zhao L., Yu D., Meng K., Yang P. (2022). *Lactobacillus plantarum* FRT4 Alleviated Obesity by Modulating Gut Microbiota and Liver Metabolome in High-Fat Diet-Induced Obese Mice. Food Nutr. Res..

[31] Koren S., Walenz B.P., Berlin K., Miller J.R., Bergman N.H., Phillippy A.M. (2017). Canu: Scalable and Accurate Long-Read Assembly via Adaptive k-Mer Weighting and Repeat Separation. Genome Res..

[32] Hyatt D., Chen G.-L., Locascio P.F., Land M.L., Larimer F.W., Hauser L.J. (2010). Prodigal: Prokaryotic Gene Recognition and Translation Initiation Site Identification. BMC Bioinform..

[33] Tarailo-Graovac M., Chen N. (2009). Using RepeatMasker to Identify Repetitive Elements in Genomic Sequences. Curr. Protoc. Bioinform..

[34] Chan P.P., Lowe T.M. (2019). tRNAscan-SE: Searching for tRNA Genes in Genomic Sequences. Methods Mol. Biol..

[35] Nawrocki E.P., Eddy S.R. (2013). Infernal 1.1: 100-Fold Faster RNA Homology Searches. Bioinformatics.

[36] Bland C., Ramsey T.L., Sabree F., Lowe M., Brown K., Kyrpides N.C., Hugenholtz P. (2007). CRISPR Recognition Tool (CRT): A Tool for Automatic Detection of Clustered Regularly Interspaced Palindromic Repeats. BMC Bioinform..

[37] Bertelli C., Brinkman F.S.L. (2018). Improved Genomic Island Predictions with IslandPath-DIMOB. Bioinformatics.

[38] Akhter S., Aziz R.K., Edwards R.A. (2012). PhiSpy: A Novel Algorithm for Finding Prophages in Bacterial Genomes That Combines Similarity- and Composition-Based Strategies. Nucleic Acids Res..

[39] Blin K., Shaw S., Steinke K., Villebro R., Ziemert N., Lee S.Y., Medema M.H., Weber T. (2019). antiSMASH 5.0: Updates to the Secondary Metabolite Genome Mining Pipeline. Nucleic Acids Res..

[40] Krzywinski M., Schein J., Birol I., Connors J., Gascoyne R., Horsman D., Jones S.J., Marra M.A. (2009). Circos: An Information Aesthetic for Comparative Genomics. Genome Res..

[41] Zhou Z., Luo M., Zhang H., Yin Y., Cai Y., Zhu Z.-J. (2022). Metabolite Annotation from Knowns to Unknowns through Knowledge-Guided Multi-Layer Metabolic Networking. Nat. Commun..

[42] Davray D., Bawane H., Kulkarni R. (2023). Non-Redundant Nature of *Lactiplantibacillus plantarum* Plasmidome Revealed by Comparative Genomic Analysis of 105 Strains. Food Microbiol..

[43] Wardman J.F., Bains R.K., Rahfeld P., Withers S.G. (2022). Carbohydrate-Active Enzymes (CAZymes) in the Gut Microbiome. Nat. Rev. Microbiol..

[44] Carpi F.M., Coman M.M., Silvi S., Picciolini M., Verdenelli M.C., Napolioni V. (2022). Comprehensive Pan-Genome Analysis of *Lactiplantibacillus plantarum* Complete Genomes. J. Appl. Microbiol..

[45] Zhang Z., Niu H., Qu Q., Guo D., Wan X., Yang Q., Mo Z., Tan S., Xiang Q., Tian X. (2025). Advancements in *Lactiplantibacillus plantarum*: Probiotic Characteristics, Gene Editing Technologies and Applications. Crit. Rev. Food Sci. Nutr..

[46] Kok C.R., Rose D., Hutkins R. (2023). Predicting Personalized Responses to Dietary Fiber Interventions: Opportunities for Modulation of the Gut Microbiome to Improve Health. Annu. Rev. Food Sci. Technol..

[47] White B.A., Lamed R., Bayer E.A., Flint H.J. (2014). Biomass Utilization by Gut Microbiomes. Annu. Rev. Microbiol..

[48] Raba G., Luis A.S. (2023). Mucin Utilization by Gut Microbiota: Recent Advances on Characterization of Key Enzymes. Essays Biochem..

[49] Peng Z., Ehrmann M.A., Waldhuber A., Niemeyer C., Miethke T., Frick J.-S., Xiong T., Vogel R.F. (2017). Phosphotransferase Systems in *Enterococcus faecalis* OG1RF Enhance Anti-Stress Capacity in Vitro and in Vivo. Res. Microbiol..

[50] Quazi F., Molday R.S. (2011). Lipid Transport by Mammalian ABC Proteins. Essays Biochem..

[51] van Hoek M.L., Hoang K.V., Gunn J.S. (2019). Two-Component Systems in Francisella Species. Front. Cell. Infect. Microbiol..

[52] Groisman E.A. (2016). Feedback Control of Two-Component Regulatory Systems. Annu. Rev. Microbiol..

[53] Zheng W., Song H., Luo Z., Wu H., Chen L., Wang Y., Cui H., Zhang Y., Wang B., Li W. (2021). Acetylcholine Ameliorates Colitis by Promoting IL-10 Secretion of Monocytic Myeloid-Derived Suppressor Cells through the nAChR/ERK Pathway. Proc. Natl. Acad. Sci. USA.

[54] Ananth M.R., Rajebhosale P., Kim R., Talmage D.A., Role L.W. (2023). Basal Forebrain Cholinergic Signalling: Development, Connectivity and Roles in Cognition. Nat. Rev. Neurosci..

[55] Zapata-Pérez R., Wanders R.J.A., van Karnebeek C.D.M., Houtkooper R.H. (2021). NAD+ Homeostasis in Human Health and Disease. EMBO Mol. Med..

[56] Wohl J., Petersen M. (2020). Phenolic Metabolism in the Hornwort Anthoceros Agrestis: 4-Coumarate CoA Ligase and 4-Hydroxybenzoate CoA Ligase. Plant Cell Rep..

[57] Zhang G., Lian Y., Li Q., Zhou S., Zhang L., Chen L., Tang J., Liu H., Li N., Pan Q. (2025). Vagal Pathway Activation Links Chronic Stress to Decline in Intestinal Stem Cell Function. Cell Stem Cell.

[58] Hosomi K., Saito M., Park J., Murakami H., Shibata N., Ando M., Nagatake T., Konishi K., Ohno H., Tanisawa K. (2022). Oral Administration of *Blautia wexlerae* Ameliorates Obesity and Type 2 Diabetes via Metabolic Remodeling of the Gut Microbiota. Nat. Commun..

[59] Xiao W., Wang R.-S., Handy D.E., Loscalzo J. (2018). NAD(H) and NADP(H) Redox Couples and Cellular Energy Metabolism. Antioxid. Redox Signal.

[60] Myakala K., Wang X.X., Shults N.V., Krawczyk E., Jones B.A., Yang X., Rosenberg A.Z., Ginley B., Sarder P., Brodsky L. (2023). NAD Metabolism Modulates Inflammation and Mitochondria Function in Diabetic Kidney Disease. J. Biol. Chem..

[61] Abdellatif M., Sedej S., Kroemer G. (2021). NAD+ Metabolism in Cardiac Health, Aging, and Disease. Circulation.

[62] Yaku K., Nakagawa T. (2023). NAD+ Precursors in Human Health and Disease: Current Status and Future Prospects. Antioxid. Redox Signal.

[63] Chini C.C.S., Cordeiro H.S., Tran N.L.K., Chini E.N. (2024). NAD Metabolism: Role in Senescence Regulation and Aging. Aging Cell.

[64] Strefeler A., Blanco-Fernandez J., Jourdain A.A. (2024). Nucleosides Are Overlooked Fuels in Central Carbon Metabolism. Trends Endocrinol. Metab..

[65] Idzko M., Ferrari D., Riegel A.-K., Eltzschig H.K. (2014). Extracellular Nucleotide and Nucleoside Signaling in Vascular and Blood Disease. Blood.

[66] Kaur T., Weadick B., Mace T.A., Desai K., Odom H., Govindarajan R. (2022). Nucleoside Transporters and Immunosuppressive Adenosine Signaling in the Tumor Microenvironment: Potential Therapeutic Opportunities. Pharmacol. Ther..

[67] Proffitt C., Bidkhori G., Lee S., Tebani A., Mardinoglu A., Uhlen M., Moyes D.L., Shoaie S. (2022). Genome-Scale Metabolic Modelling of the Human Gut Microbiome Reveals Changes in the Glyoxylate and Dicarboxylate Metabolism in Metabolic Disorders. iScience.

[68] Chen G., Ye G., Zhang X., Liu X., Tu Y., Ye Z., Liu J., Guo Q., Wang Z., Wang L. (2018). Metabolomics Reveals Protection of Resveratrol in Diet-Induced Metabolic Risk Factors in Abdominal Muscle. Cell. Physiol. Biochem..

[69] Prochownik E.V., Wang H. (2021). The Metabolic Fates of Pyruvate in Normal and Neoplastic Cells. Cells.

[70] Li Z., Zhang H. (2016). Reprogramming of Glucose, Fatty Acid and Amino Acid Metabolism for Cancer Progression. Cell. Mol. Life Sci..

[71] Liao Y., Chen Q., Liu L., Huang H., Sun J., Bai X., Jin C., Li H., Sun F., Xiao X. (2024). Amino Acid Is a Major Carbon Source for Hepatic Lipogenesis. Cell Metab..

[72] Asakura J., Nagao M., Shinohara M., Hosooka T., Kuwahara N., Nishimori M., Tanaka H., Satomi-Kobayashi S., Matsui S., Sasaki T. (2025). Impaired Cardiac Branched-Chain Amino Acid Metabolism in a Novel Model of Diabetic Cardiomyopathy. Cardiovasc. Diabetol..

